# Dr. John H. Miller

**Published:** 1901-01

**Authors:** 


DR. JOHN H. MILLER, of San Leandro, Cal., U. of B. '77, died at his home October 12, 1900. Dr. Miller had been in active practice until a short time before his death.

